# Observation-derived 2010-2019 trends in methane emissions and intensities from US oil and gas fields tied to activity metrics

**DOI:** 10.1073/pnas.2217900120

**Published:** 2023-04-17

**Authors:** Xiao Lu, Daniel J. Jacob, Yuzhong Zhang, Lu Shen, Melissa P. Sulprizio, Joannes D. Maasakkers, Daniel J. Varon, Zhen Qu, Zichong Chen, Benjamin Hmiel, Robert J. Parker, Hartmut Boesch, Haolin Wang, Cheng He, Shaojia Fan

**Affiliations:** ^a^School of Atmospheric Sciences, Sun Yat-sen University, Southern Marine Science and Engineering Guangdong Laboratory (Zhuhai), Zhuhai, Guangdong 519082, China; ^b^Guangdong Provincial Observation and Research Station for Climate Environment and Air Quality Change in the Pearl River Estuary, Zhuhai, Guangdong Province 519082, China; ^c^Key Laboratory of Tropical Atmosphere-Ocean System (Sun Yat-sen University), Ministry of Education, Zhuhai, Guangdong Province 519082, China; ^d^School of Engineering and Applied Sciences, Harvard University, Cambridge, MA 02138; ^e^Key Laboratory of Coastal Environment and Resources of Zhejiang Province, School of Engineering, Westlake University, Hangzhou, Zhejiang Province 310024, China; ^f^Institute of Advanced Technology, Westlake Institute for Advanced Study, Hangzhou, Zhejiang Province 310024, China; ^g^Department of Atmospheric and Oceanic Sciences, School of Physics, Peking University, Beijing 100871, China; ^h^SRON Netherlands Institute for Space Research, Utrecht, the Netherlands; ^i^Department of Marine, Earth, and Atmospheric Sciences, North Carolina State University, Raleigh, NC 27695; ^j^Environmental Defense Fund, Washington, DC 20009; ^k^National Centre for Earth Observation, Space Park Leicester, University of Leicester, Leicester LE1 7RH, UK; ^l^Department of Physics and Astronomy, Earth Observation Science, University of Leicester, Leicester LE1 7RH, UK

**Keywords:** methane, oil/gas emission, inversion, decadal trends, production activity

## Abstract

The United States is the world’s largest oil/gas methane emitter according to current national reports. Reducing these emissions is a top priority in the US government’s climate action plan. Here, we use a 2010 to 2019 high-resolution inversion of surface and satellite observations of atmospheric methane to quantify emission trends for individual oil/gas production regions in North America and relate them to production and infrastructure. We estimate a mean US oil/gas methane emission of 14.8 (12.4 to 16.5) Tg a^−1^ for 2010 to 2019, 70% higher than reported by the US Environmental Protection Agency. While emissions in Canada and Mexico decreased over the period, US emissions increased from 2010 to 2014, decreased until 2017, and rose again afterward. Increases were driven by the largest production regions (Permian, Anadarko, Marcellus), while emissions in the smaller production regions generally decreased. Much of the year-to-year emission variability can be explained by oil/gas production rates, active well counts, and new wells drilled, with the 2014 to 2017 decrease driven by reduction in new wells and the 2017 to 2019 surge driven by upswing of production. We find a steady decrease in the oil/gas methane intensity (emission per unit methane gas production) for almost all major US production regions. The mean US methane intensity decreased from 3.7% in 2010 to 2.5% in 2019. If the methane intensity for the oil/gas supply chain continues to decrease at this pace, we may expect a 32% decrease in US oil/gas emissions by 2030 despite projected increases in production.

## Significance

The United States accounts for a large share of global methane emissions from the oil/gas industry. Analysis of satellite and surface observations of atmospheric methane reveals larger-than-reported year-to-year variability of 2010 to 2019 US oil/gas methane emissions. This variability reflects trends in oil/gas production rates, number of active wells, and drilling of new wells. Emissions surged after 2017 as production increased. The methane intensity from the US oil/gas industry (methane emitted per unit methane gas produced) decreased steadily after 2010. Extension of this decreasing trend to 2030 (target date of the Global Methane Pledge) would result in a 32% decrease in US oil/gas methane emissions and 15% decrease in total anthropogenic emissions relative to 2019 despite an increase in production.

Atmospheric methane (CH_4_) is a powerful climate forcer accounting for a third of the global temperature rise since the preindustrial era (1). It has a much shorter lifetime than carbon dioxide (CO_2_) and 80 times higher warming potential over a 20-y horizon. Mitigation of methane emissions is critical for limiting global warming within 1.5 °C and also has cobenefits for public health and food productivity (2). Methane has a range of sources including wetlands as the major natural emitter, and agriculture (livestock, rice), waste (landfills, wastewater), and fossil fuel exploitation (coal, oil, gas) as the main anthropogenic emitters (3). Curbing methane emissions from the oil/gas industry is of particular interest due to its high feasibility and economic benefit (4–8).

The United States is the leading oil/gas methane emitter in the world according to United Nations Framework Convention on Climate Change (UNFCCC) reports, with a national emission of 8.1 Tg a^−1^ that accounts for 15% of global oil/gas methane emissions for 2019 (9). Oil and gas production in the United States increased by 137% and 88%, respectively, from 2005 to 2019 (10, 11). The US Environmental Protection Agency (EPA) reports no significant change in its methane emission inventory over that period, reflecting improved industry practices and capture of associated gas to offset increasing oil production (12). However, top-down estimates from observations of atmospheric methane indicate 0.4 to 6% a^−1^ increases in US oil/gas methane emissions over the 2006 to 2017 period (13–17) and national emissions about twice higher than given by EPA (5, 15, 16, 18, 19). Insufficient accounting of anomalously large sources (the so-called superemitters) has been blamed for at least part of the inventory underestimate (5, 20, 21), but there has been little study of the factors driving the long-term emission trend.

Here, we conduct an inverse analysis of 2010 to 2019 methane observations from satellite and surface sites over North America to determine the annual trends of emissions for different oil/gas production regions over that period. We relate the emission trends to activity metrics to identify the dominant drivers of oil/gas methane emissions. We also report trends in methane intensities, defined as the fraction of gas emitted to the atmosphere rather than taken to market, as an indicator of industry practices and of the potential to decrease emissions in the future.

## Results

### Top-Down 2010 to 2019 Estimates of Oil and Gas Methane Emissions.

**Fig. 1.**
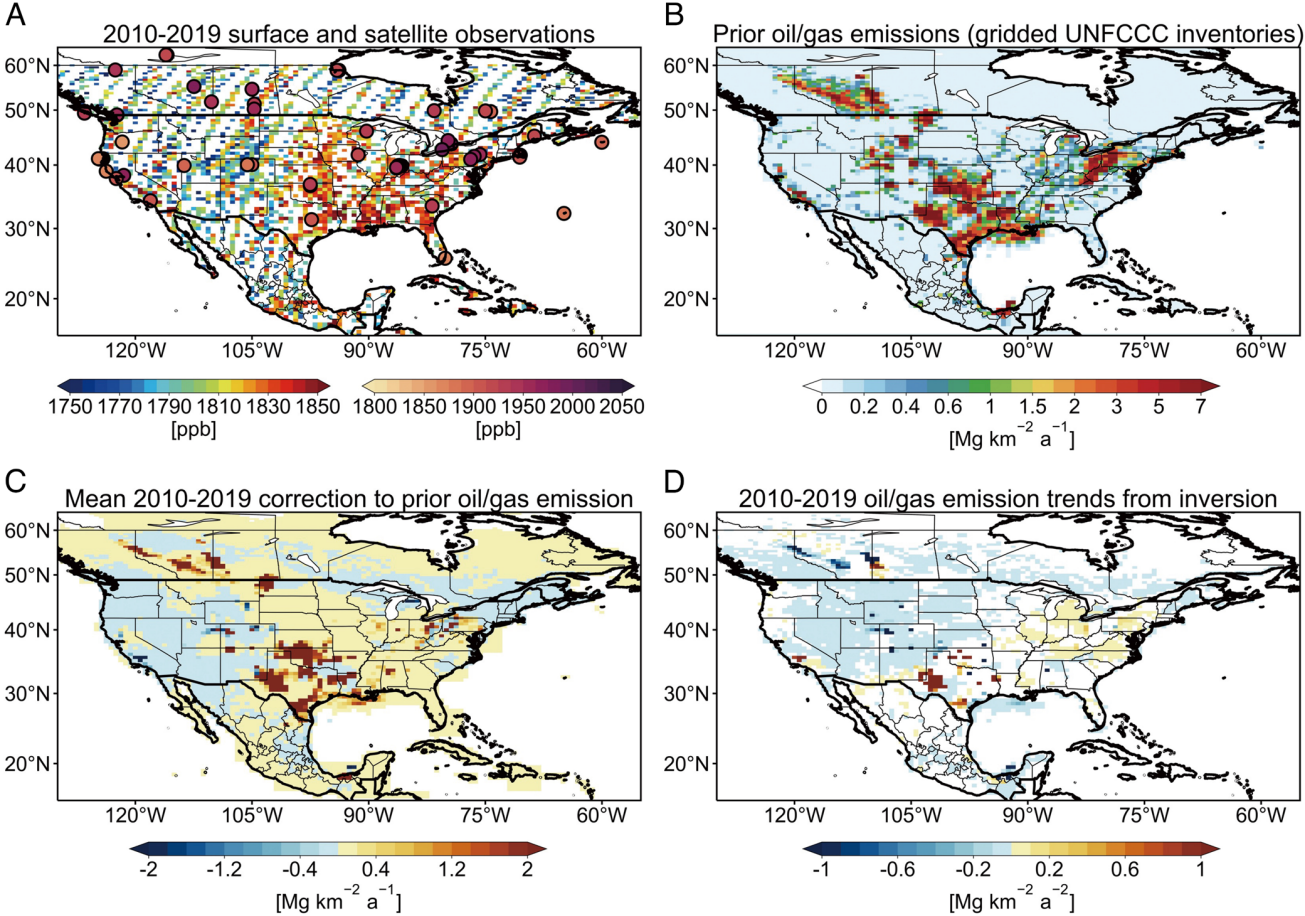
Application of satellite and surface observations of atmospheric methane to quantify oil/gas emissions and 2010 to 2019 trends. Panel *A* shows mean observed surface methane mixing ratios from in situ surface and tower observations archived in the GLOBALVIEWplus CH4 ObsPack data product (circles), and dry column mixing ratios retrieved from the GOSAT satellite instrument and averaged on the 0.5° × 0.625° inversion grid. Panel *B* shows prior oil/gas emissions for the inversion from the spatially gridded versions of the US, Canada, and Mexico official national inventories reported to the United Nations Framework Convention on Climate Change (UNFCCC). Panel* C* shows the posterior corrections to mean 2010 to 2019 oil/gas methane emissions from the inversion, and Panel *D* shows the 2010 to 2019 linear trends in oil/gas emissions. The linear trends are fitted by ordinary least-squares linear regression to the inversion results for individual years. Only trends with *P-value *≤0.34 are shown.

We quantify 2010 to 2019 annual methane emissions from the oil/gas industry by inverse analysis of atmospheric methane observations from the Greenhouse Gases Observing Satellite (GOSAT) satellite instrument (22) and the GLOBALVIEWplus CH_4_ ObsPack dataset of surface (including tower) sites (23) (Fig. 1*A*), making use of the complementarity between the two observation platforms (15, 24–26). We use the continental-scale GEOS-Chem chemical transport model (27) at 0.5° × 0.625° resolution as forward model in the inversion to relate emissions to concentrations. The inversion strategy follows our previous work which examined national methane emissions from all sources for 2010 to 2017 (15), but here we extend it to 2019 with focus on the oil/gas sector and individual production regions. Emissions are optimized by drawing information from the observations and prior estimates following the Bayesian rule, where the prior estimates are from gridded versions of the national anthropogenic inventories reported to the UNFCCC (28–30) together with WetCHARTs v1.3.1 (31) for wetlands (Fig. 1*B* and *SI Appendix*, Fig. S1). The inversion is done for individual years in 2010 to 2019, updating boundary conditions over the oceans in each year with a consistent global inversion for 2010 to 2019 (25, 32, 33). The same prior emissions in North America are used for all years, effectively assuming no trend as a prior assumption. The posterior (optimal) solution for emissions on the 0.5° × 0.625° grid is obtained analytically to yield closed-form error statistics and information content, and to enable the construction of an ensemble of solutions using different inversion parameter assumptions. Posterior emission estimates from a 12-member inversion ensemble with each reporting two estimates by different sectoral attribution methods define the uncertainty range on the posterior results (*SI Appendix*, Table S1). See *Materials and Methods* for more details on inversion procedures, evaluation of posterior emissions, and uncertainty analyses.

**Fig. 2.**
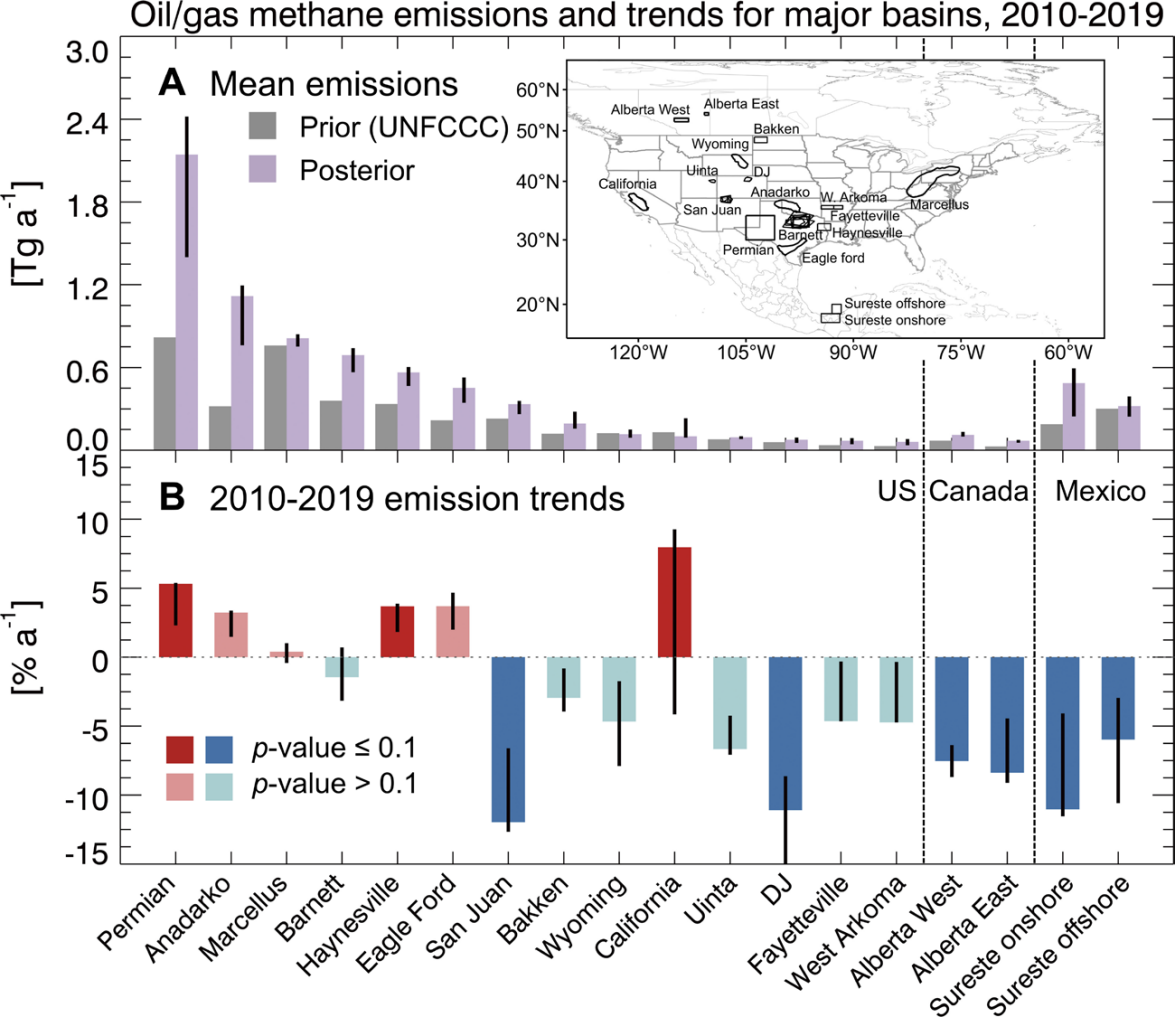
Mean oil/gas methane emissions and trends for major production regions in North America, 2010 to 2019. Panel *A* shows the posterior emissions from the inversion compared to the gridded UNFCCC reports. The *Inset* panel defines the individual regions. Panel *B* shows the 2010 to 2019 emission trends inferred by ordinary least-squares linear regression on the posterior emissions for individual years. The vertical bars represent the uncertainty ranges derived from the 12-member inversion ensemble with each generating two emission estimates based on different source attribution methods, resulting in 24 estimates in total.

The inversion returns yearly posterior gridded correction factors to the prior emission estimates on the 0.5° × 0.625° grid (*SI Appendix*, Fig. S2). Fig. 1*C* shows the mean 2010 to 2019 corrections for the oil/gas emission sector, based on the contribution from that sector to total prior emissions in each grid cell combined with error statistics by sector following Shen et al. (34). The inversion is able in this manner to separate oil/gas emissions and trends from those of other sectors (*Materials and Methods*). It has difficulty in separating oil and gas emissions for some regions and therefore we report combined oil/gas emissions. Fig. 2*A* quantifies oil/gas methane emissions for the 18 major production regions in North America. The 14 US regions account for, respectively, 60% and 80% of the 2019 national oil and gas production, and we will see later that they largely determine the year-to-year trend in the national oil/gas emissions.

We find significant underestimation in the national inventories of 2010 to 2019 oil/gas methane emissions across North America, prominently in the central-south and midwestern US, Alberta and Saskatchewan in Canada, and the Sureste onshore oil field in Mexico. Exceptions are the Marcellus field, which is exclusively of gas production, and the Sureste offshore oil field. These results are consistent with previous top-down studies (15, 16, 19, 34–37). Our best posterior estimate of US oil/gas methane emission averaged over 2010 to 2019 is 14.8 (12.4 to 16.5 from the inversion ensemble) Tg a^−1^, 70% higher than the most recent EPA estimate of 8.7 Tg a^−1^ for the same period (12). Our best posterior estimates for Canada and Mexico are 2.6 (2.2 to 3.3) and 1.2 (0.8 to 1.4) Tg a^−1^, 67% and 50% higher, respectively, than their national reports. Our inversion results for individual production regions in Fig. 2*A* are generally consistent with reported estimates from field campaigns covering different time periods in 2010 to 2019 and also with an inversion from the higher-resolution TROPOspheric Monitoring Instrument (TROPOMI) satellite instrument available for 2018 to 2019 (*SI Appendix*, Table S2). Discrepancies may partly reflect differences in observing periods. Our estimate of 2010 to 2019 emissions from the Permian (2.1 Tg a^−1^) is low compared to other top-down estimates for the post-2017 period (2.3 to 3.7 Tg a^−1^) (19, 37–40) which may reflect in part our use of an EPA prior estimate of 0.8 Tg a^−1^ known to be too low (19, 37) and our longer time horizon.

### Interpretation of 2010 to 2019 Trends in US Oil/Gas Methane Emissions.

We now examine the annual trends of oil/gas methane emissions over the 2010 to 2019 period as informed by our inversion for individual years. Fig. 1*D* shows the spatial distributions of the long-term trends as obtained by ordinary least-squares linear regression, and Fig. 2*B* gives the trends for the 18 major oil/gas production regions. Trends in oil/gas emissions can be clearly separated by the inversion from trends in emissions from other sectors (*SI Appendix*, Fig. S3). We find that oil/gas emissions over the 2010 to 2019 period changed by +7% for the United States, −23% for Canada, and −60% for Mexico. There are large spatial differences in trends between US production regions. The top six US production regions with the largest emissions including the Permian, Anadarko, Marcellus, Haynesville, and Eagle Ford show increasing trends in 2010 to 2019 ranging from 0.4 to 5% a^−1^, except for the Barnett which shows a 1.5% a^−1^ decrease. Other regions with smaller emissions generally show emission decreases. The decreasing trends in Canada may reflect the implementation of the Pan-Canadian Framework on Clean Growth and Climate Change for reducing methane released from the oil/gas sector (41). The decreasing trends in Mexico may reflect increasing utilization of associated gas from oil production (42).

**Fig. 3.**
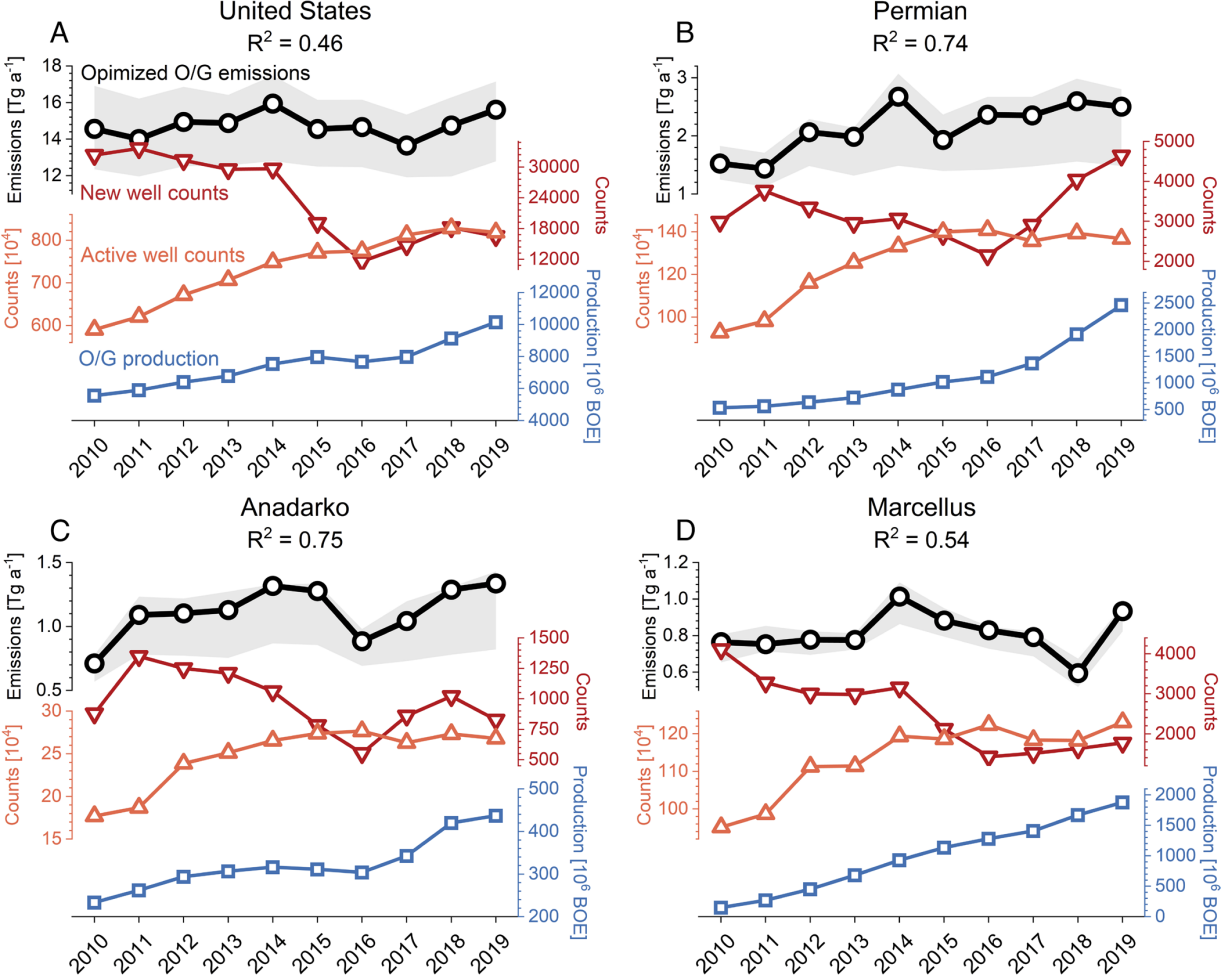
2010 to 2019 trends in oil/gas (O/G) methane emissions in the United States. Posterior emission estimates from the inversion are shown for the contiguous United States (Panel *A*) and for the three major emission regions of the Permian, Anadarko, and Marcellus (Panels *B*, *C*, and *D*) (Fig. 2). Gray shadings represent the range from the inversion ensemble; *SI Appendix*, Fig. S4 shows the trends from each ensemble member to demonstrate the consistency of the year-to-year variability across the ensemble. Also shown are trends in oil/gas production (in unit of barrel of oil equivalent, BOE), count of active wells, and count of new wells (first reported production year) from the Enverus DrillingInfo (44). These three variables are used in a multiple linear regression model to fit the posterior oil/gas emissions, with the coefficient of determination (*R^2^*) shown as *Inset*. *SI Appendix*, Table S3 gives the detailed results for the regression model. Results for smaller oil/gas production regions are shown in *SI Appendix*, Fig. S5.

Fig. 3*A* shows the year-to-year trends in US oil/gas methane emissions over the 2010 to 2019 period as optimized by the base inversion. US oil/gas emissions increased from 14.6 Tg a^−1^ in 2010 to 15.9 Tg a^−1^ in 2014, decreased to 13.6 Tg a^−1^ in 2017, and rose again to 15.6 Tg a^−1^ in 2019. This year-to-year variability is consistent across the inversion ensemble (*SI Appendix*, Fig. S4). The US EPA inventory (12) has considerably less interannual variability (8.7 ± 0.1 Tg a^−1^, mean ± SD for 2010 to 2019). Previous top-down studies reported large oil/gas methane emission increases for the United States of 6% a^−1^ for 2008 to 2014 (43) and 3.4% a^−1^ for 2005 to 2014 (14), whereas we find 2.4% a^−1^ for 2010 to 2014. Maasakkers et al. (16) reported an increase of only 0.4% a^−1^ for 2010 to 2015, which we explain by the steep drop from 2014 to 2015. Lu et al. (15) reported an increase in oil and decrease in gas emissions for 2010 to 2017 but no significant trends for combined oil/gas emissions. The post-2017 emission surge has not been reported before to our knowledge.

We find that the interannual variability in the US oil/gas emissions can be largely explained by that in the 14 oil/gas production regions shown in Fig. 2 (*R*^2^ = 0.74), and half can be explained by the top six regions with the largest emissions (*R*^2^ = 0.50). We show yearly emissions for the top three production regions (Permian, Anadarko, and Marcellus) in Fig. 3 and others in *SI Appendix*, Fig. S5. The US oil/gas emission increase in 2010 to 2014 was largely driven by the Permian where emissions increased from 1.5 Tg a^−1^ in 2010 to 2.7 Tg a^−1^ in 2014 (Fig. 3*B*). The Anadarko (increasing by 0.7 Tg a^−1^) and the Marcellus (increasing by 0.3 Tg a^−1^) also contributed. Previous reports of large emissions in the Permian focused on the post-2017 period when TROPOMI observations became available (20, 37, 38, 40). Our inversion shows that Permian emissions were already at current high values by 2014. The national decrease in oil/gas emissions for the 2014 to 2017 period reflects a combination of trends in the Permian, Anadarko, and Marcellus (Fig. 3), while the post-2017 rebound is largely driven by the Anadarko, Marcellus, Barnett, and Haynesville. The net near-zero decadal trend of US emission in 2010 to 2019 obfuscates these subdecadal swings and spatial differences between oil/gas production regions as shown in Fig. 2*B*.

Fig. 3 further shows the relationship between the year-to-year variability in oil/gas emissions and different activity metrics. Here, we use three metrics: i) oil/gas production rate, ii) counts of active wells, and iii) counts of new wells drilled from the Enverus Drilling Info database (44). Previous aircraft-based surveys have shown strong spatial correlation of emissions with gas production and active well pad count in the Fayetteville Shale (45). Oil/gas production relates to methane emissions as high production tends to increase the number of operating facilities and the gas flowing through them. In addition, increase in production may challenge the capacity of midstream infrastructure to manage the gas flow (46). Omara et al. (47) showed that wells with low production can contribute a large proportion of oil/gas emissions, indicating that active well count number should be another predictor of oil/gas methane emissions. New wells are prone to high methane emissions due to uncontrolled emissions from exploratory drillings, a spike at well completion, and decreasing tank flash emissions during the first year of operation (48). We find that the number of new wells shows a strong correlation with the annual crude oil price from the West Texas Intermediate Cushing (WTI-Cushing) oil benchmark (*r *= 0.93) (49) and with the natural gas price from the Henry hub (https://www.eia.gov/dnav/ng/hist/rngwhhdA.htm) of (*r *= 0.75) (50). Takeaway capacity is also a possible predictor for oil/gas methane emissions as shown in a recent study of weekly emissions in Permian (51), but data are unavailable for 2010 to 2019.

We find that the three metrics of production rates, active well counts, and new well counts are complementary and together can explain 46% of the year-to-year variability of total US oil/gas emissions over the 2010 to 2019 period (Fig. 3*A*), as derived from a multiple linear regression (*SI Appendix*, Table S3). The explanatory power is higher for individual production regions, typically 60 to 80% (Fig. 3 and *SI Appendix*, Fig. S5), although the importance of each metric varies by region indicating differences in operating practices. The 2010 to 2014 increase in US oil/gas emissions by 9% was associated with a rise in oil/gas production by 34%, a rise in the number of active wells by 27%, and a sustained drilling of new wells of more than 30,000 a^−1^ over the period. All major oil/gas production regions showed similar behavior. The 2014 to 2017 drop in US oil/gas methane emissions was associated with a 60% reduction in new well development, while total oil/gas production and number of active wells remained stable. Decline in new well development was found in all major production regions (Fig. 3). This was likely driven by the drop of annual crude oil price by about 50% over the period (49).

The recent 2017 to 2019 emission surge appears to be driven by the revival of US oil/gas production which increased by 30% in this period. The number of active wells and new wells was 8% higher in 2017 to 2019 than the 2015 to 2016 mean, reflecting the upswing of oil price. The rise in oil/gas production was mostly in the Anadarko, Marcellus, and Haynesville (Fig. 3 and *SI Appendix*, Fig. S5), which accounted for most of the emission increase. Post-2017 emission increases in the Permian were weak despite large increases in oil/gas production and new well development, and this could reflect an increase in pipeline takeaway capacity (51).

### Decreasing Methane Intensity from US Oil/Gas Production.

**Fig. 4.**
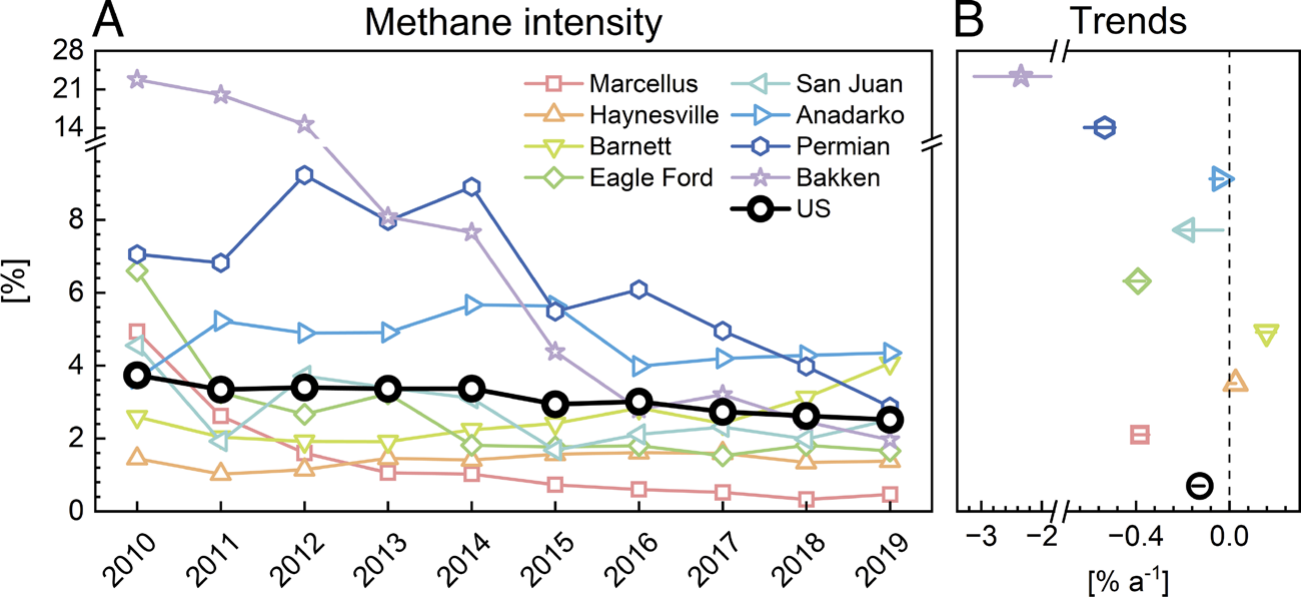
2010 to 2019 methane intensity from the US oil/gas industry. Methane intensity is defined as the total oil/gas emission per unit of gas produced. It represents the amount of methane emitted rather than used for fuel (United States) or taken to market (production regions). Panel *A* shows the year-to-year variability. Trends in Panel *B* are obtained by ordinary least-squares linear regression. Horizontal bars show the ranges from the inversion ensemble.

Fig. 4 and *SI Appendix*, Table S4 show the magnitudes and trends of methane intensity, defined as methane emission integrated along the oil and gas supply chain per unit of methane gas production. This definition follows the US Environmental Defense Fund (EDF) (5, 46) and a number of previous studies (42, 52–54). It is similar for production regions to the methane intensity defined by the Oil and Gas Climate Initiative as upstream oil/gas emissions (from production, processing, and storage) per unit of gas marketed, since upstream emissions dominate in production regions (55). The methane intensity effectively measures the potential for reducing emissions from the oil/gas industry by marketing methane rather than emitting it. Some studies report methane intensity normalized by combined oil and gas production to estimate the amount of gas emitted per unit of total energy produced by oil/gas (40, 47, 56). The two definitions of methane intensity show similar trends (*SI Appendix*, Fig. S6). We use the first definition of methane intensity in what follows.

We derive a mean methane intensity from the US oil/gas industry of 3.1% averaged over 2010 to 2019 assuming an average methane content of 90% by volume (5). The 2010 to 2019 mean methane intensities for the eight largest US production regions (with 2010 to 2019 mean oil/gas production >100 million barrel of oil equivalent (BOE) and emission >0.2 Tg a^−1^) vary from 1.4 to 8.8%, using reported values of methane content in natural gas for individual regions (5). The Bakken and Permian show the largest methane intensities of 8.8% and 6.3%, respectively. Both are mainly oil-producing regions where much of the by-produced gas may be vented or inefficiently flared rather than marketed (57). In comparison, gas-dominated regions such as the Marcellus, Haynesville, and Fayetteville have much lower methane intensity of less than 1.5%, reflecting a stronger motivation for these regions to capture the gas for marketing.

We find a steady decrease in the US oil/gas methane intensity of −0.13% a^−1^ (*P* < 0.01) (relative annual reduction of −0.43% a^−1^), from 3.7% in 2010 to 2.5% in 2019. The 2017 to 2019 emission surge was driven by a large increase in production despite a continued decrease in methane intensity. 6 of the 8 largest oil/gas production regions shown in Fig. 4 have decreasing trends in methane intensity (Fig. 4*B*). Among the six smaller production regions, Denver-Julesburg also shows a decreasing trend with *P* < 0.01, while the others show insignificant trends (*SI Appendix*, Table S4). The Bakken and Permian show large methane intensity decreases of −2.3% a^−1^ and −0.53 % a^−1^, respectively, effectively narrowing the spread of methane intensity across production regions.

The decreasing methane intensity in the United States and in the major oil/gas production regions reflects a slower increase or a decrease in oil/gas methane emissions relative to the increase in oil/gas production. We find that the 2010 to 2019 oil/gas methane intensity trends from all the 14 US regions of Fig. 2 are negatively correlated with their respective trends in oil/gas production (*r *= −0.6) and oil/gas production per well (*r *= −0.4) (*SI Appendix*, Table S4). Production regions with wells that are more mature and productive tend to leak less methane per unit production. Other small production regions show decreasing methane intensity even with decreasing oil/gas production and less productive wells (San Juan and Uinta).

Beyond the dominant role of production trends, we suggest that an additional driver for the decreasing methane intensity may be the US EPA’s implementation of new source performance standards (NSPS) for the oil/gas sector. The NSPS proposed in 2011 (finalized form in 2012) tightened emission standards for a range of production facilities and processes including completions of hydraulically fractured gas wells, and pneumatic controllers and storage tanks from oil and gas wells (58, 59). The rules were reinforced in 2015 (finalized form in 2016) with emission standards for additional facilities and processes including hydraulically fractured oil well completions, fugitive emissions from well sites, and compressor stations (59, 60). These NSPS rules affected facilities that were constructed or modified after the date of the original proposals (2011 and 2015).

The Bakken and Permian had large methane intensities in 2010 to 2014 (8% or higher). They both show large decreases in methane intensity in 2015 (Fig. 4*A*) when the NSPS targeted emissions from the new oil production sector (60). Schneising et al. (40) previously reported a significant drop of methane intensity in Bakken from 2009 to 2011 to 2018 to 2019 and suggested that the trend may be driven by industry initiatives for leak detection and repair, replacement or upgrade of high-emitting devices, and reduction of venting or flaring. For the Permian, the US EPA’s Greenhouse Gas Reporting Program indicates a 21% increase in gathering pipeline miles from its first report year in 2016 to 2019 (61), and the pipeline takeaway capacity increased by ~10% from 2018 to 2019 (earlier data are not available), indicating more effective gas capture for marketing (51). This may explain the flat emission in the Permian despite large increases in oil/gas production after 2016.

Gas-dominated production regions such as the Marcellus and Haynesville have much lower methane intensity than the Bakken and Permian and also show decreases in methane intensity over 2010 to 2019. Decreasing methane intensity in the Marcellus, the leading shale gas production region in the United States, can likely be attributed to reduced new well drilling in the second half of the decade and new regulations requiring capture of gas from the completion‐venting step of hydraulic fracturing (62), though we see a rebound of new wells drilled and methane emissions in 2019 (Fig. 3*D*).

The Anadarko and Barnett stand out as the production regions with the largest methane intensities in 2019 and no significant decreases over the 2010 to 2019 period. Barnett is a mature shale production region with few new wells drilled in 2010 to 2019 (52). It shows an increase of methane intensity over 2017 to 2019 for reasons that are unclear. Persistent high methane intensity in Anadarko is consistent with findings from a previous study (40). These two regions would be attractive targets for decreasing methane emissions.

## Discussion

Even with the overall decreasing trend in 2010 to 2019 methane intensity, the United States is still emitting a large amount of oil/gas methane concentrated in a few major production regions and with no sign of an actual emission decrease since production continues to increase. Our best estimate of 15.6 Tg a^−1^ for US oil/gas emissions in 2019, compared to the US EPA estimate of 8.7 Tg a^−1^, increases the United States’contribution to global oil/gas methane emission from 15% to 28% if based on UNFCCC reports for other countries (9). The United States is committed with 121 other countries to the Global Methane Pledge, an initiative to reduce collective methane emissions by 30% below 2020 levels by 2030 (63). The US Methane Emission Reduction Action has prioritized new actions to reduce methane leaked from the oil/gas industry (64). At the same time, the US Energy Information Administration projects an increase in oil and gas production by 18% and 13%, respectively, in 2030 relative to 2020 levels in the International Energy Outlook (IEO) 2022 Reference Scenario (65). If the 2010 to 2019 decreasing trend in methane intensity shown in Fig. 4 continues at its current rate (relative annual reduction of −0.43% a^−1^), the methane intensity would drop to 1.5% by 2030. Applying this methane intensity to the increased production in the IEO 2022 Reference Scenario and our best emission estimate of 15.6 Tg a^−1^ for 2019 indicate an US oil/gas emission of 10.6 Tg a^−1^ in 2030, 32% lower than 2019 levels, and a 15% decrease in total US anthropogenic emissions if other sectors taken from the US EPA inventory are assumed constant (12). Sustaining such a continued decrease in methane intensity may be a challenge as oil/gas fields approach maturity and wells become less productive, as is evident in the present-day Barnett (52), and development of new oil/gas fields would likely cause the methane intensity to increase. New efforts to decrease the methane intensity from oil/gas production, as outlined in the US Methane Emission Reduction Action (64), will be necessary to meet the United States’contribution to the Global Methane Pledge.

## Materials and Methods

### Observations of Atmospheric Methane.

#### In-situ observations.

In-situ methane measurements are from the GLOBALVIEWplus CH_4_ ObsPack product compiled by the National Oceanic and Atmospheric Administration (NOAA) Global Monitoring Laboratory (23). We use daily daytime (10 to 16 local time) methane mixing ratio at surface and tower measurement sites with continuous 10-y records in 2010 to 2019 over North America, composing a total of 73,297 data points from 47 sites.

#### GOSAT satellite retrievals.

We use dry column methane mixing rations (XCH_4_) in 2010 to 2019 from the GOSAT satellite instrument produced by the University of Leicester version 9.0 Proxy XCH_4_ retrieval (22). We exclude glint data over the oceans and poleward of 60° due to seasonally biased sampling and potentially high errors. We obtain a total of 243233 GOSAT retrievals for 2010 to 2019 over North America.

### Bottom-Up Emissions Used as Prior Estimates for the Inversion.

We use gridded versions of the national anthropogenic methane emission inventories for the United States, Canada, and Mexico reported to the UNFCCC. Spatial allocation of these emissions by sector on a 0.1° × 0.1° grid was done by Maasakkers et al. (28) for 2012 US emissions based on the 2016 US EPA Greenhouse Gas Inventory, by Scarpelli et al. (29) for 2018 Canada emissions based on the 2020 Environment and Climate Change Canada report, and by Scarpelli et al. (30) for 2015 Mexico emissions based on the 2018 Instituto Nacional de Ecologia y Cambio Climatico report. We use the same anthropogenic emissions as prior estimates for all years in the 2010 to 2019 period, so that emission trends from the inversion are solely driven by observations.

Wetland methane emissions (*SI Appendix*, Fig. S1*D*) are from the mean of the nine highest-performance members of the WetCHARTs v1.3.1 inventory ensemble at 0.5° × 0.5° resolution (31), selected for their fit to the global GOSAT inversion results (33). We use 2010 to 2019 mean emissions by month as prior estimates in the inversion to avoid introducing prior information on interannual variability. Open fire emissions are daily values for individual years from the Global Fire Emissions Database version 4s (66). Small constant natural emissions are from Etiope et al. (67) scaled to Hmiel et al. (68) for seepages and from Fung et al. (69) for termites.

### GEOS-Chem Forward Model Simulation.

We use the nested version of the GEOS-Chem 12.5.0 chemical transport model (https://doi.org/10.5281/zenodo.3403111) to simulate the atmospheric methane concentrations and their sensitivity to methane emissions. The model is driven by MERRA-2 reanalysis meteorological fields (70). We conduct model simulations at 0.5° × 0.625° resolution over the North America domain (130-55°W, 15-65°N) for each individual year of 2010 to 2019, with the initial and boundary conditions at the edge of the domain archived from a global model simulation using posterior methane emissions optimized from a global inversion of GOSAT satellite observations (25, 32, 33). The boundary conditions capture the global trend of methane concentrations over the 2010 to 2019 period but may have errors in interannual variability. We therefore choose to optimize the boundary conditions in the four directions (east, west, south, and north) for individual years as state vector elements in the inversion.

### Atmospheric Inverse Analysis.

The inversion procedure including the design of state vector, error estimates, and optimization strategy mostly follows Lu et al. (15). We use a Gaussian mixture model (71) to generate 600 Gaussian emission functions defined by location, spread, and magnitude in the prior gridded emissions, in order to preserve high (0.5° × 0.625°) resolution for regions with strong localized emissions while smoothing the solution in regions of weak emissions. The state vector x is then defined as the emission from each of the 600 Gaussians, plus the correction to the model boundary conditions as described earlier, for a total dimension n=604.

We solve the optimal estimate of x by minimizing the Bayesian cost function $J\left(x\right)$:

[1] $$\begin{array}{c} J\left(x\right)={\left(x-x_{A}\right)}^{T}{S_{A}}^{-1}\left(x-x_{A}\right)+\gamma {\left(y-Kx\right)}^{T}{S_{O}}^{-1}\left(y-Kx\right), \end{array}$$

where $x_{A}$ is the prior estimate of x , $S_{A}$ is the prior error covariance matrix, y is the observation vector, $S_{O}$ is the observation error covariance matrix, K=∂y/∂x is the Jacobian matrix representing the sensitivity of modeled methane concentrations to emissions, and γ is a regularization factor to prevent overfitting. Minimizing Eq. **1** at $\nabla_{x}J\left(x\right)=0$ yields an analytical solution for the posterior state vector $\hat{x}$ , its error covariance matrix $\hat{S}$ , and the averaging kernel matrix A:

[2] $$\hat{x}=x_{A}+{\left(\gamma K^{T}{S_{O}}^{-1}K+{S_{A}}^{-1}\right)}^{-1}\gamma K^{T}{S_{O}}^{-1}\left(y-Kx_{A}\right),$$

[3] $$\hat{S}={\left(\gamma K^{T}{S_{O}}^{-1}K+{S_{A}}^{-1}\right)}^{-1},$$

[4] $$A=\frac{\partial \hat{x}}{\partial x}=I_{n}-\hat{S}{S_{A}}^{-1}.$$

The inversion returns the posterior estimates of mean emissions and averaging kernel sensitivities for each Gaussian, and these values can then be mapped back to the 0.5° × 0.625° grid space.

We construct **K,** $S_{A}$**,** $S_{O}$ and the regularization factor γ following Lu et al. (15). Our base inversion assumes log-normal error distribution for the prior emission magnitude of each Gaussian with a geometric SD of 2 (corresponding to a factor of 2 uncertainty). This allows us to avoid unphysical negative posterior emissions (72) and to better capture the heavy tail of the emission distribution (5, 7, 21, 73) as compared to previous studies assuming normal error distributions.

### Evaluation of Posterior Estimate.

We evaluate the inversion results by comparing the ability of GEOS-Chem simulations with posterior versus prior emissions to fit the observed GOSAT methane columns, the GLOBALVIEWplus CH_4_ ObsPack surface/tower observations of methane concentrations, and independent ground-based methane column observations at three sites from the Total Carbon Column Observing Network (TCCON). *SI Appendix*, Fig. S7 shows that the posterior simulation with optimized emissions and trends significantly reduces the model mean bias in US surface and tower measurements from −11 ppb in the prior simulation to −6 ppb, and the rms error (rmse) from 22 to 15 ppb. We find that there is no decadal trend in the model bias relative to both in situ and GOSAT observations in the posterior simulations. The model is biased high at the three TCCON sites and this is mostly driven by the Lamont, Oklahoma site, but again there is no bias in the trend.

### Attributing Posterior Emissions and Trends to Emission Sectors.

Our inversion returns posterior correction factors ( $f_{0}$ ) to the total methane emissions in individual 0.5° × 0.625° grid cells and for individual years. We apply two methods to allocate $f_{0}$ to correction factors *f_i_* for individual sectors *i* in that grid cell. The first method (base estimate) derives $f_{i}$ based on the fraction of sectoral emissions to the total prior emissions in the grid cell and the error statistics for that sector given in the prior US EPA emission inventory (28), following Shen et al. (34). The second method assumes that the prior sectoral distribution of emissions in the grid cell is correct and that the posterior scaling factors apply equally to all sectors in the grid cell ( $f_{i}=f_{0}$).

We examined the ability of the inversion to quantify oil/gas emissions in individual production regions separately from other sources (such as livestock) in those regions. This was done by transforming the posterior full-dimension state vector $\hat{x}$ to a reduced state vector ${\hat{x}}_{red}$ , with sectoral methane emissions aggregated over the defined region as elements. We can then use the corresponding posterior error covariance matrix ${\hat{S}}_{red}$ to quantify the ability of the inversion to separate emissions from different sectors within the region. Further details on this approach are in the study by Maasakkers et al. (74). We find that we can successfully separate oil/gas emissions from other sectors in the United States and in most of the major oil/gas production regions as indicated by the small posterior error correlation coefficients for all sector pairs (*SI Appendix*, Fig. S8). However, separating oil from gas emissions can be challenging for some regions and we only report combined oil/gas methane emissions.

### Uncertainty of the Posterior Estimates.

Our analytical inversion returns the closed-form posterior error covariance matrix $\hat{S}$ (Eq. **3**) which can be used to examine the uncertainty of the posterior emissions. However, $\hat{S}$ does not reflect the uncertainty in the inversion parameters. We derive an alternative estimate of the uncertainty based on the range of posterior emissions from a 24-member inversion ensemble including different forms and values of $S_{A}$ , different values of the regularization parameter γ , and different sectoral attribution methods (*SI Appendix*, Table S1). Generation of this ensemble is immediate since all members use the same Jacobian matrix **K**. *SI Appendix*, Fig. S9 compares the uncertainties estimated from $\hat{S}$ and from the inversion ensemble for the year 2015. We find that the emission uncertainty defined by the range of the inversion ensemble is generally larger than the error inferred from the diagonal of $\hat{S}$ except for small production regions, consistent with the finding in the study by Chen et al. (75). We therefore mainly use the 24-member range in the inversion ensemble to characterize uncertainty, but report uncertainty from $\hat{S}$ where applicable (e.g., when describing a single inversion result or when uncertainty from $\hat{S}$ is larger than the ensemble range).

*SI Appendix*, Fig. S4 shows the range of posterior oil/gas emissions from the 24-member ensemble (*SI Appendix*, Table S1) in the United States and the three largest basins (Permian, Anadarko, and Marcellus). We find that assuming a log-normal error distribution (inversions #1-6) for prior emission rather than a normal distribution (inversions #7-12) typically results in higher posterior emission estimates, by better capturing the observed heavy tail of the emission probability density functions. Assuming a larger prior error allows stronger upward correction of oil/gas emissions. Using a source-dependent $f_{i}$ attributes more upward correction to oil/gas emissions as the oil sector has larger uncertainty in the gridded EPA emission inventory (28). Reducing the weight of GOSAT observations decreases the ability to optimize methane emissions but has relatively little impact on the magnitude. The year-to-year variability is in general consistent across the inversion ensemble.

## Supplementary Material

Appendix 01 (PDF)

## Data Availability

Data (.csv/.nc/.sav) have been deposited in GitHub (https://github.com/luxiaoatchemsysu/Data-USoilgasCH4) (76).
