# Supplementary material for: Observation-derived 2010-2019 trends in methane emissions and intensities from US oil and gas fields tied to activity metrics
Source: Proc Natl Acad Sci U S A. 2023 Apr 17;120(17):e2217900120. doi: 10.1073/pnas.2217900120 (PMC10151460; doi:10.1073/pnas.2217900120)
Supplement: Supplementary file 1 — Appendix 01 (PDF) [file pnas.2217900120.sapp.pdf]

PNAS

Supporting Information for

Observation-derived 2010-2019 trends in methane emissions and intensities from US oil and gas fields tied to activity metrics

Xiao Lu[a,b,c], Daniel J. Jacob[d,1], Yuzhong Zhang[e,f], Lu Shen[g], Melissa P. Sulprizio[d], Joannes D. Maasakkers[h], Daniel J. Varon[d], Zhen Qu[i], Zichong Chen[d], Benjamin Hmiel[j], Robert J. Parker[k,l], Hartmut Boesch[k,l], Haolin Wang[a], Cheng He[a], and Shaojia Fan[a,b,c]

[a]School of Atmospheric Sciences, Sun Yat-sen University, Southern Marine Science and Engineering Guangdong Laboratory (Zhuhai), Zhuhai, Guangdong 519082, China

[b]Guangdong Provincial Observation and Research Station for Climate Environment and Air Quality Change in the Pearl River Estuary, Zhuhai, Guangdong Province 519082, China

[c]Key Laboratory of Tropical Atmosphere-Ocean System (Sun Yat-sen University), Ministry of Education, Zhuhai, Guangdong Province 519082, China

[d]School of Engineering and Applied Sciences, Harvard University, Cambridge, MA 02138

[e]Key Laboratory of Coastal Environment and Resources of Zhejiang Province, School of Engineering, Westlake University, Hangzhou, Zhejiang Province 310024, China

[f]Institute of Advanced Technology, Westlake Institute for Advanced Study, Hangzhou, Zhejiang Province 310024, China

[g]Department of Atmospheric and Oceanic Sciences, School of Physics, Peking University, Beijing 100871, China

[h]SRON Netherlands Institute for Space Research, Utrecht, the Netherlands

[i]Department of Marine, Earth, and Atmospheric Sciences, North Carolina State University, Raleigh, NC 27695

[j]Environmental Defense Fund, Washington, DC 20009

[k]National Centre for Earth Observation, Space Park Leicester, University of Leicester, Leicester LE1 7RH,United Kingdom

[l]Department of Physics and Astronomy, Earth Observation Science, University of Leicester , Leicester LE1 7RH,United Kingdom

Correspondence to: Xiao Lu; Daniel J. Jacob
Email: luxiao25@mail.sysu.edu.cn; djacob@fas.harvard.edu

This PDF file includes:

Figures S1 to S9
Tables S1 to S4

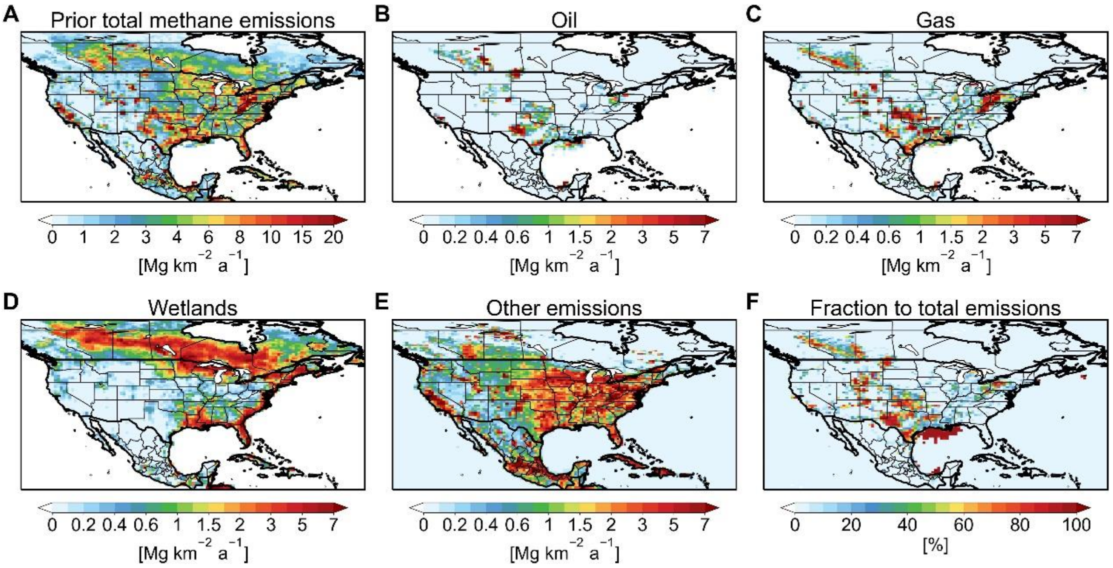

Figure S1. Prior emissions used in the inversion. Prior methane emissions are shown for (A) all sectors, (B) oil, (C) gas, (D) wetlands, and (E) other emissions. Panel (F) shows the fraction of oil/gas emissions to total methane emissions. Anthropogenic emissions are from spatially gridded versions of the US, Canada, and Mexico official national inventories. Wetland emissions are from the mean of the high-performance subset of the WetCHARTs inventory ensemble.

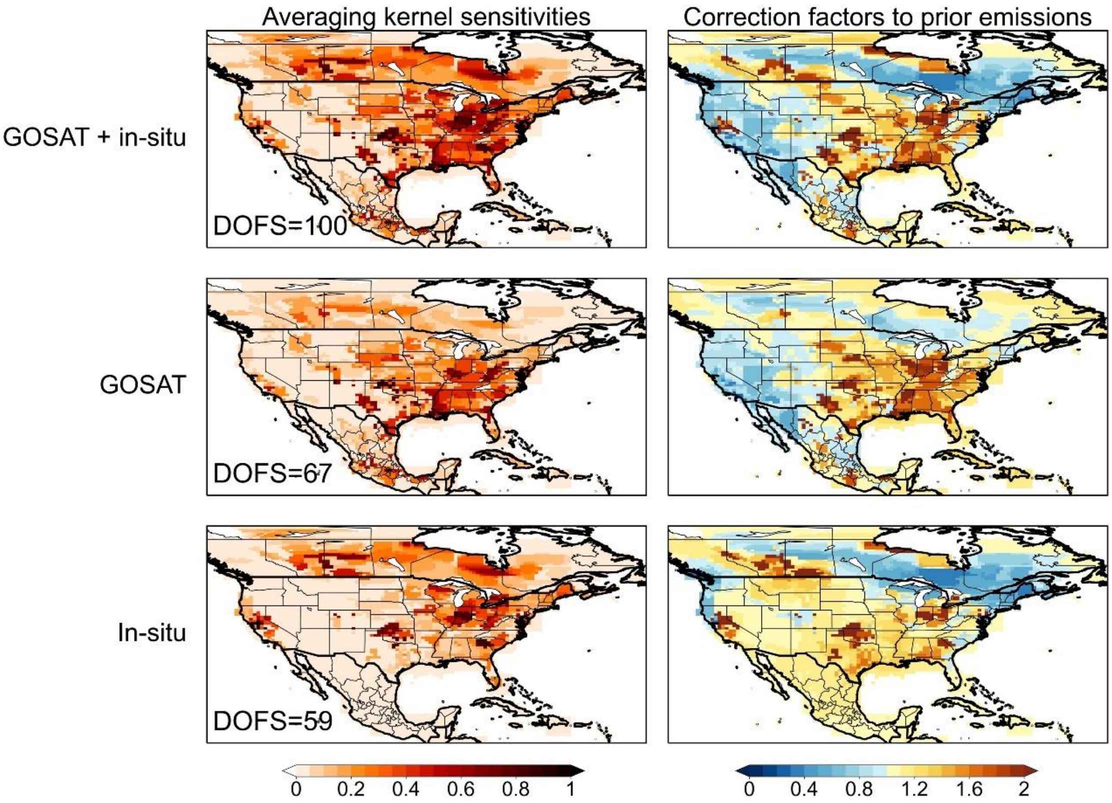

Figure S2. Optimization of mean 2010–2019 methane emissions over North America. Results are from the base inversion using both GOSAT and GLOBALVIEWplus in situ observations, the GOSAT-only inversion, and the in-situ-only inversion. The left panels show the mean averaging kernel sensitivities (diagonal elements of the averaging kernel matrix). The degrees of freedom for signal (DOFS, defined as the trace of the averaging kernel matrix) are shown in the inset. The right panels show the posterior correction factors, i.e., the multiplicative factors applied to the total prior emissions in Fig. S1A.

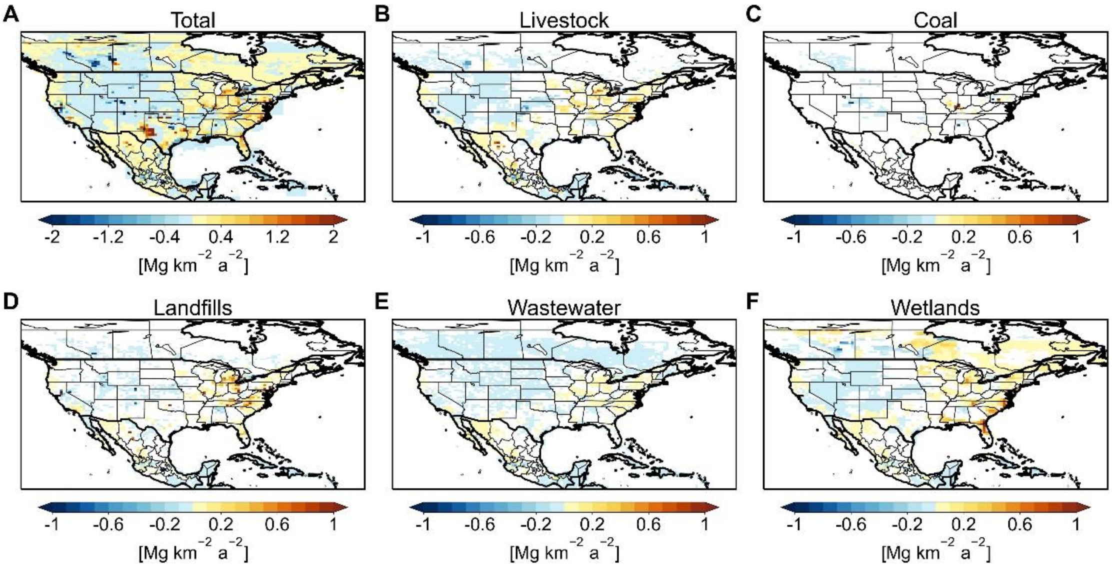

Fig. S3 2010–2019 linear trends in methane emissions. The linear trends are fitted by linear regression to the inversion results for individual years.

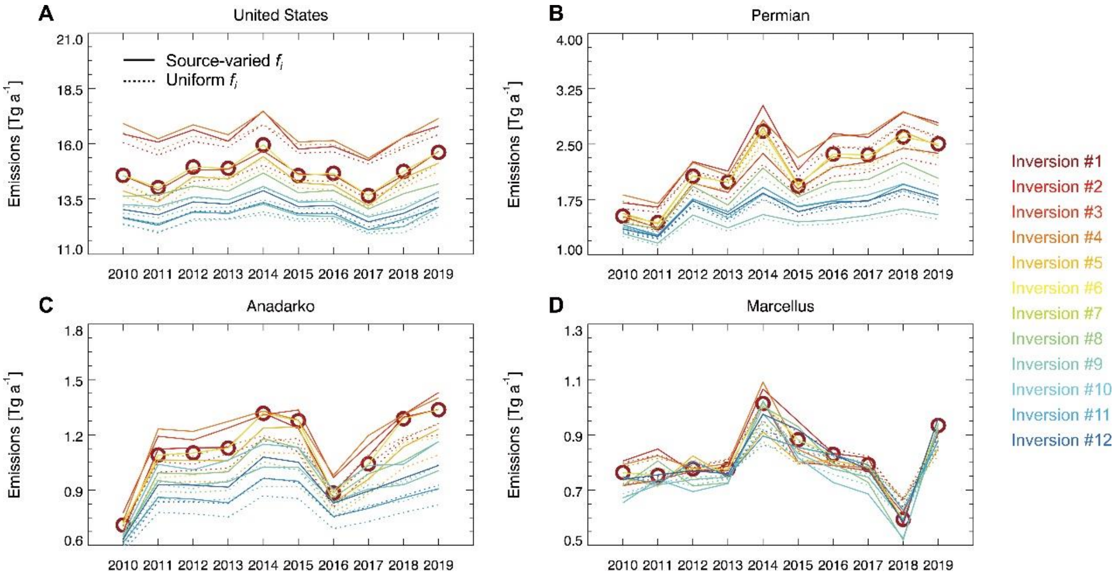

Fig. S4 2010-2019 trends in oil/gas methane emissions in the US, Permian, Anadarko, and Marcellus from inversion ensemble. Each line represents an inversion result using different parameters. The red circles highlight the results from the base inversion (Inversion #1).

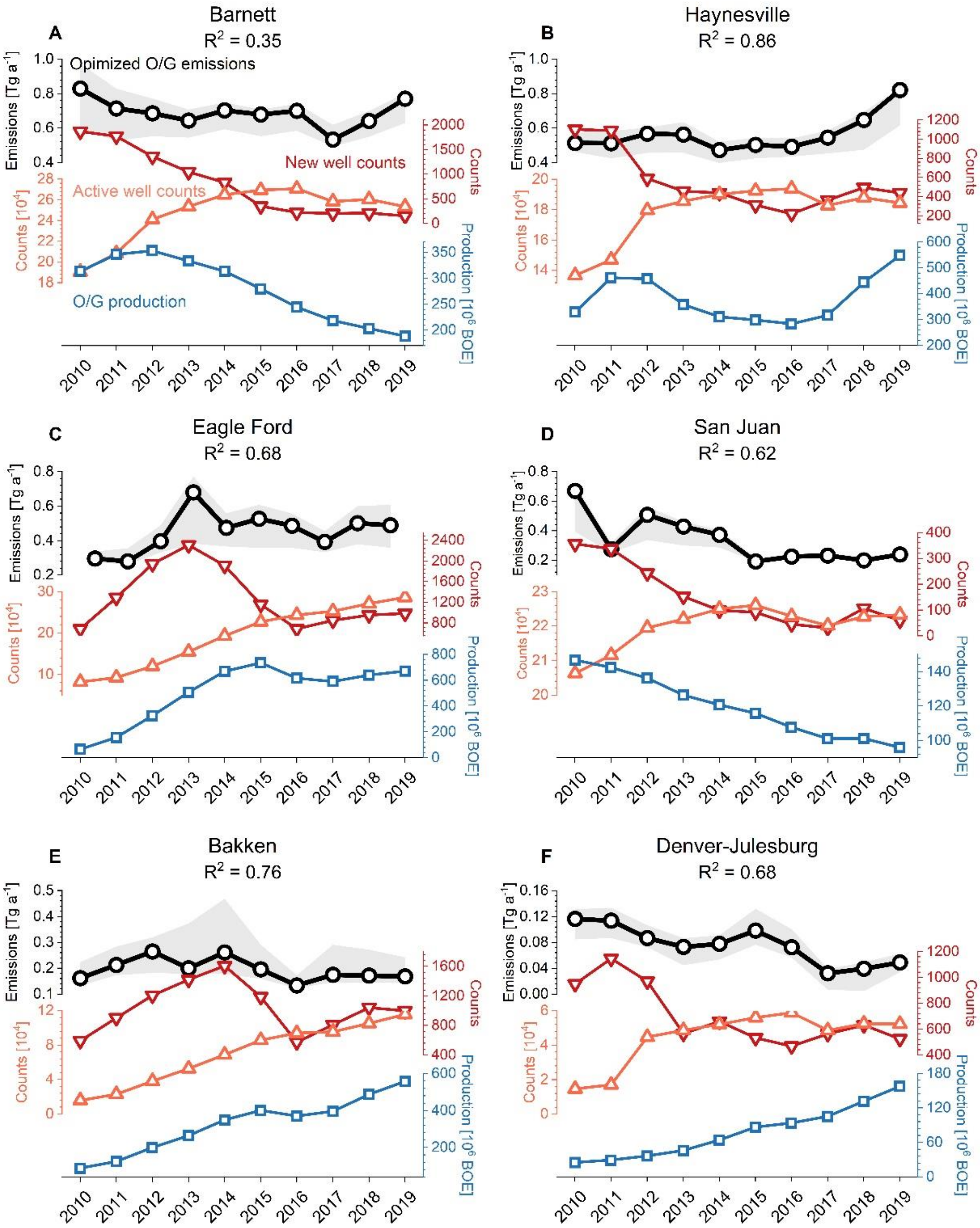

Fig. S5 Same as Fig.3 but for different oil/gas production regions.

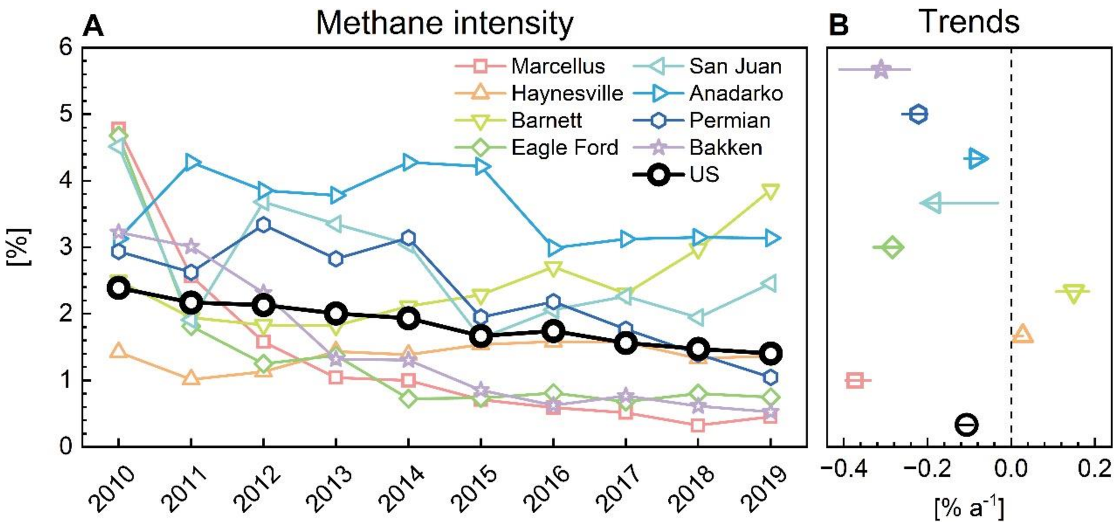

Fig. S6 Same as Fig.4 but for methane intensity defined as oil/gas methane emissions normalized by the combined oil and gas production based on energy content, assuming one barrel of oil has the same amount of energy content as 6,000 cubic feet of natural gas.

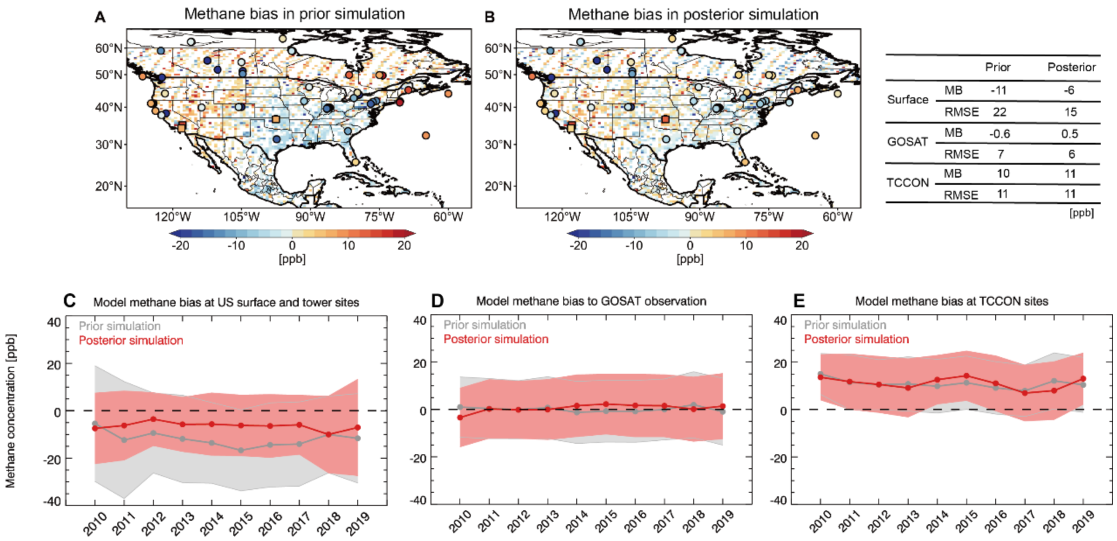

Fig. S7 Evaluation of the posterior simulation to fit surface and tower, GOSAT, and TCCON observations for 2010–2019. Panels A and B show the mean differences between GEOS-Chem simulations and the observations using either prior or posterior methane emissions. Panels C-E show the model bias relative to surface and tower, GOSAT, and TCCON observations averaged for each year. The shadings represent the standard deviation of the bias.

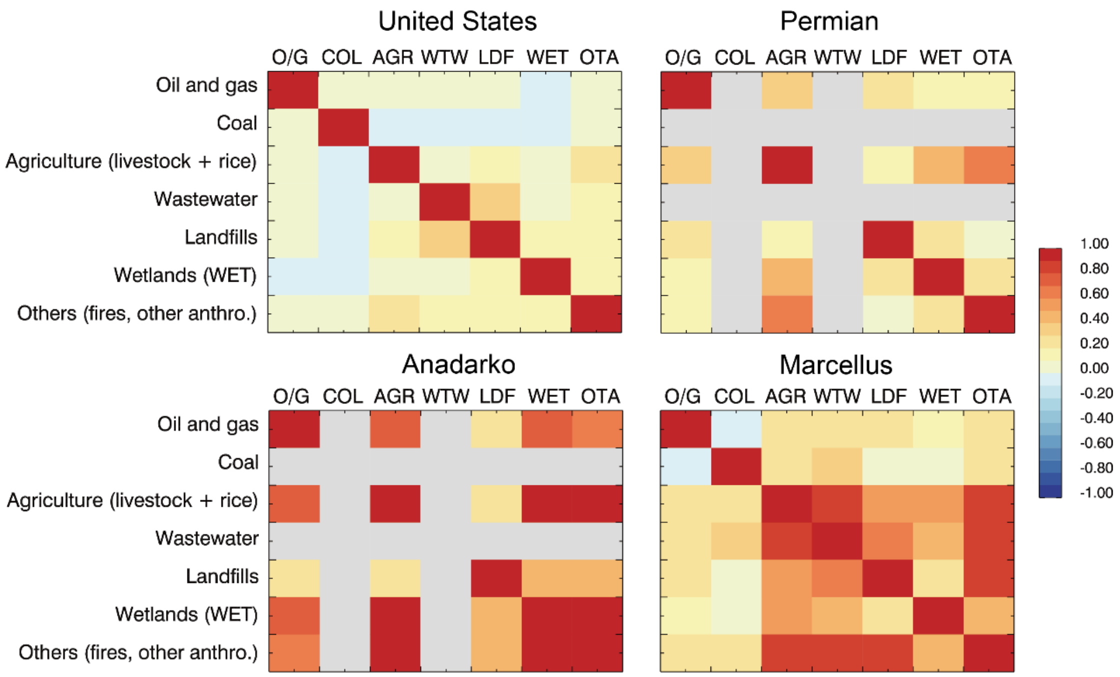

Fig. S8 Ability of the inversion to separate posterior methane emission sectors. The figure shows posterior error correlation coefficients (*r*) between sectoral methane emissions in the US (CONUS) and three major oil/gas production regions, using the sector-aggregated error covariance matrix as described in Method. Error correlation coefficients indicate the ability of the inversion to separate emissions between sectors (0:perfectly, ±1: not at all). Grey shadings indicate that there is no emission from this sector in the region. Results are from the base inversion for the year 2015. Results for other years show similar patterns.

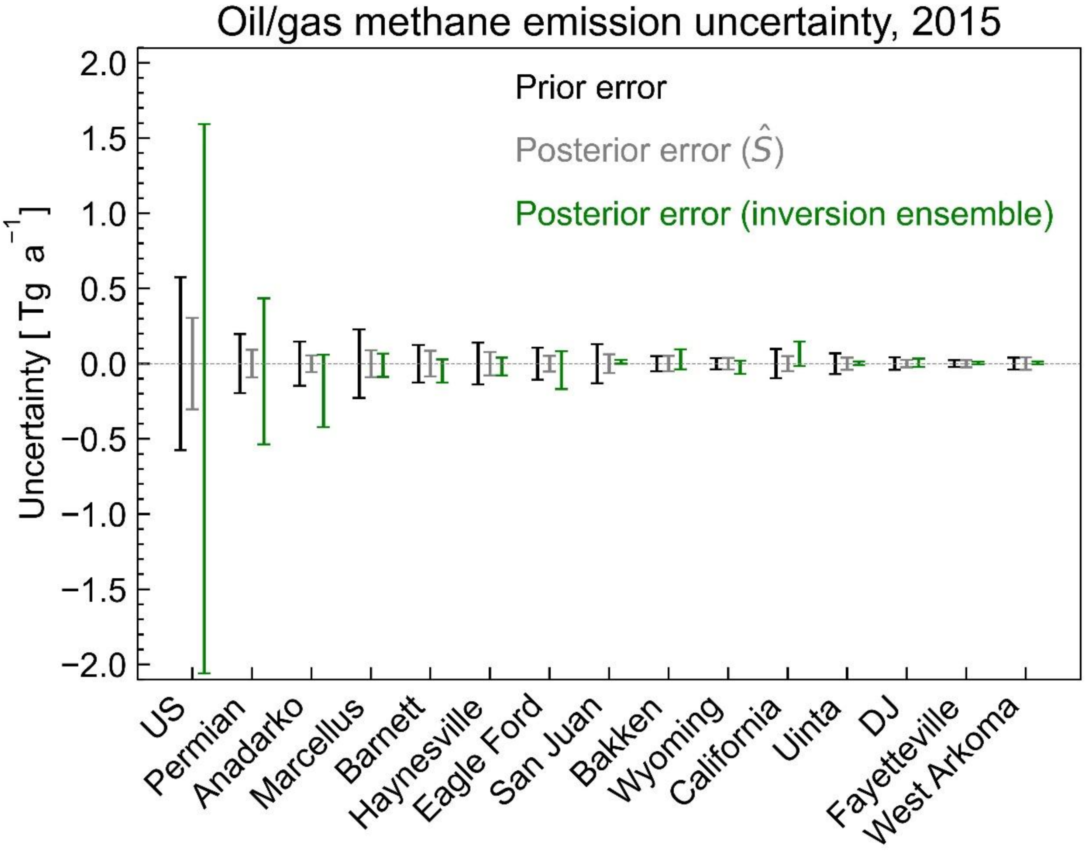

Figure S9. Comparison of uncertainty estimates in the prior and posterior oil/gas emissions over the US and individual production regions from derived from the posterior error covariance $\hat{S}$ and the inversion ensemble.

Table S1. Settings for generation of the 12-member inversion ensemble yielding 24 estimates of oil/gas emissions [a].

Inversion member	Regularization parameter γ		Prior error standard deviation distribution for emission	Prior error standard deviation value for emission	Prior error standard deviation value for boundary conditions [ppb]
	In situ	GOSAT			
1 (Base)	1.0	1.0	Lognormal	50%	10
2	1.0	1.0	Lognormal	75%	10
3	1.0	0.5	Lognormal	50%	10
4	1.0	0.5	Lognormal	75%	10
5	1.0	1.0	Lognormal	quadrature sum [b]	10
6	1.0	1.0	Lognormal	50%	5
7	1.0	1.0	Normal	50%	10
8	1.0	1.0	Normal	75%	10
9	1.0	0.5	Normal	50%	10
10	1.0	0.5	Normal	75%	10
11	1.0	1.0	Normal	quadrature sum	10
12	1.0	1.0	Normal	50%	5

[a] Settings different to the base inversion are underlined. For each inversion, we apply two methods to allocate the posterior correction factor of total methane emissions to the oil/gas sector, as introduced in *Methods*, so the 12-member inversion ensemble yields 24 estimates of oil/gas emissions.

[b] Adding the errors from individual sectors in quadrature following Maasakkers et al. (2021).

[c] Prior estimate of emissions from the oil/gas emissions in Permian was increased by a factor of 4 (-3.2 Tg a^{-1}) from the EPA inventory, reflecting previous evidence that the EPA inventory is too low.

Table S2. Field and inversion estimates of oil and gas emissions (Gg a^{-1}) in different US production regions.

Region	Oil/gas emissions (Mg year^{-1})						
	Field campaigns vs this inversion					TROPOMI-based inversion vs this inversion	
	**Reference**	**Year of measurement**	**Field estimates**[a]	**Bottom-up estimates by Alvarez et al.**[b] **for 2015**	**This study** [c]	**TROPOMI-inversion estimates** [d]	**This study** [e]
Bakken	Peischl et al. (2016) (1)	2014	237 (123-342)	210 (149-306)	266 (177-467)	123 (91-155)	168 (144-240)
Barnett	Karion et al. (2015) (2)	2013	526 (430-622)	499 (430-577)	648 (553-707)	478 (412-540)	770 (630-802)
DJ	Petron et al. (2014) (3)	2012	166 (105-227)	184	90 (72-106)	52 (29-75)	49 (33-61)
Fayetteville	Schwietzke et al. (2017) (4)	2015	237 (202-272)	201 (166-237)	42 (38-55)	36 (19-53)	59 (44-100)
Haynesville	Peischl et al.(2015) (5)	2013	639 (166-1138)	639 (420-937)	552 (457-635)	656 (551-761)	822 (616-838)
Permian	EDF(2019)	2019	2663		2503 (1483-2803)	2903 (2497-3309)	2503 (1483-2803)
San Juan	Smith et al. (2017) (6)	2015	499 (37-937)	499 (272-815)	191 (191-215)	236 (184-288)	238 (215-244)
Uinta	Karion et al. (2013) (7)	2012	482 (210-745)	254 (219-289)	95 (81-104)	96 (76-106)	60 (59-74)
West Arkoma	Peischl et al. (2015) (5)	2013	228 (0-482)	114 (65-166)	30 (20-35)	51 (36-66)	51 (36-88)
Anadarko						610 (476-744)	1335 (820-1429)
California						244 (171-317)	124 (125-186)
Eagle Ford	Peischl et al. (2018) (8)	2015	718 (578-858)		525 (357-607)	508 (417-599)	489 (357-607)
Marcellus	Ren et al. (2018) (9)	2015	668 (602-730)		881 (794-949)	613 (441-785)	934 (823-961)
Wyoming						124 (88-160)	92 (51-120)

[a] For a period of days or weeks in the year of measurement. Uncertainties are given where applicable.

[b] From Alvarez et al. (2018) (10)

[c] Yearly emissions for the corresponding data sample year. Uncertainties are given from the inversion ensemble (12 members, 24 results).

[d] From Shen et al. (2022) (11)for May 2018 to February 2020. Uncertainties are given as the 2 standard deviations (2σ) corresponding to the 95 % confidence level from Monte Carlo method.

[e] Emissions are for 2019. Uncertainties are given from the inversion ensemble (12 members, 24 results).

Table S3. Summary of the multiple linear regression model used for prediction of oil/gas methane emissions in the US[1].

Region	Coefficient of production (A)	Coefficient of active well count (B)	Coefficient of new well count (C)	Constant (D)	R^2	*p-value*
US	4.84×10^{-4} (r=0.34)	2.47×10^{-5} (r=0.17)	7.23×10^{-5} (r=0.12)	9.40	0.46	0.27
Permian	-1.67×10^{-5} (r=0.67)	2.11×10^{-2} (r=0.83)	1.41×10^{-4} (r=0.15)	-0.95	0.74	<0.05
Anadarko	1.38×10^{-3} (r=0.69)	3.07×10^{-2} (r=0.59)	4.67×10^{-4} (r=0.22)	-0.54	0.75	<0.05
Marcellus	-1.18×10^{-5} (r=0.11)	1.58×10^{-2} (r=0.37)	1.37×10^{-4} (r=0.05)	-1.32	0.54	0.17
Barnett	-1.42×10^{-3} (r=0.22)	1.53×10^{-2} (r=-0.57)	2.18×10^{-4} (r=0.48)	0.53	0.35	0.43
Haynesville	1.23×10^{-3} (r=0.80)	-7.24×10^{-2} (r=0.16)	-6.00×10^{-4} (r=-0.13)	1.72	0.86	<0.05
Eagle Ford	2.54×10^{-4} (r=0.70)	3.18×10^{-3} (r=0.48)	1.04×10^{-4} (r=0.45)	0.13	0.68	0.07
San Juan	8.59×10^{-3} (r=0.76)	-1.04×10^{-1} (r=-0.64)	-7.52×10^{-4} (r=0.70)	1.72	0.62	0.10
Bakken	-5.35×10^{-5} (r=-0.30)	-2.70×10^{-3} (r=-0.40)	1.02×10^{-4} (r=0.77)	0.12	0.76	<0.05
Wyoming	-1.42×10^{-3} (r=0.12)	1.45×10^{-2} (r=-0.57)	1.56×10^{-4} (r=0.60)	0.07	0.44	0.29
California	-1.63×10^{-4} (r=0.03)	-1.63×10^{-3} (r=0.40)	-7.88×10^{-5} (r=-0.91)	0.20	0.85	<0.05
Uinta	7.07×10^{-3} (r=0.77)	-1.36×10^{-4} (r=-0.09)	-5.80×10^{-5} (r=0.53)	-0.12	0.62	0.11
DJ	-4.24×10^{-4} (r=-0.80)	-4.85×10^{-3} (r=-0.66)	-4.12×10^{-6} (r=0.66)	0.13	0.68	0.07
Fayetteville	1.14×10^{-3} (r=-0.35)	-1.16×10^{-1} (r=-0.48)	-4.78×10^{-4} (r=0.16)	0.60	0.79	<0.05
West Arkoma	-5.35×10^{-3} (r=-0.13)	-1.41×10^{-1} (r=-0.58)	2.29×10^{-4} (r=0.32)	0.29	0.50	0.22

[1]The regression model is expressed as Emissions (in unit of Tg a^{-1}) = A×(oil/gas production, in unit of million barrels of oil equivalent (BOE)) + B×(count number of active wells) $\times10^4$+ C×(count number of new wells) + D. Correlation coefficients between each predictor and oil/gas emission are shown in the brackets.

Table S4. Summary of 2010-2019 mean oil/gas methane intensity (emission per unit methane gas production) and trends.

**Region**	**Mean methane intensity [%]**	**Mean oil/gas production [10^6 BOE]**	**Mean oil/gas production per well [10^6 BOE well^{-1}]**	**Gas fraction to total oil/gas production [%]**	**Trend in methane intensity [% a^{-1}]**	**Trend in oil/gas production [% a^{-1}]**	**Trend in oil/gas production per well [% a^{-1}]**	**Trend in gas production fraction [% a^{-1}]**
US	3.1	7498	1015	58	-0.13*	6.1*	2.4*	-1.0*
Permian	6.3	1118	861	36	-0.53*	17*	13.8*	-0.43*
Anadarko	4.7	322	1306	76	-0.04	5.9*	1.7	-1.3*
Marcellus	1.4	982	833	98	-0.38*	19	18.6*	0.05
Barnett	2.6	279	1158	95	0.16*	-6.7*	-9.8*	-0.1
Haynesville	1.4	380	2173	98	0.03	1.5	-1.6	0.05
Eagle Ford	2.6	497	2477	44	-0.40*	13*	3.4	-1.9*
San Juan	2.7	119	544	98	-0.18*	-5*	-5.7*	-0.18*
Bakken	8.8	322	4855	21	-2.34*	15*	-2.6*	1.5*
Wyoming	2.4	71	1122	64	0.10	-0.1	-5.3*	-5.4*
California	9.6	57	938	19	0.61	3.6*	-13.3	-0.6*
Uinta	3.4	32	934	78	-0.03	-4*	-10.1*	-1.9*
DJ	3.2	77	1721	50	-0.82*	19*	9.3*	-1.4*
Fayetteville	0.6	110	2849	100	0.02	-6.4	-15.3*	/
West Arkoma	5.1	13	1131	99	0.28	-8*	-11.3*	0

*denotes trend with p-value<0.1

SI References

1. Peischl J*, et al.* (2016) Quantifying atmospheric methane emissions from oil and natural gas production in the Bakken shale region of North Dakota. *J. Geophys. Res.* 121(10):6101-6111.
2. Karion A*, et al.* (2015) Aircraft-Based Estimate of Total Methane Emissions from the Barnett Shale Region. *Environ. Sci. Technol.* 49(13):8124-8131.
3. Pétron G*, et al.* (2014) A new look at methane and nonmethane hydrocarbon emissions from oil and natural gas operations in the Colorado Denver ‐ Julesburg Basin. *J. Geophys. Res.* 119(11):6836-6852.
4. Schwietzke S*, et al.* (2017) Improved Mechanistic Understanding of Natural Gas Methane Emissions from Spatially Resolved Aircraft Measurements. *Environ. Sci. Technol.* 51(12):7286-7294.
5. Peischl J*, et al.* (2015) Quantifying atmospheric methane emissions from the Haynesville, Fayetteville, and northeastern Marcellus shale gas production regions. *J. Geophys. Res.* 120(5):2119-2139.
6. Smith ML*, et al.* (2017) Airborne Quantification of Methane Emissions over the Four Corners Region. *Environ. Sci. Technol.* 51(10):5832-5837.
7. Karion A*, et al.* (2013) Methane emissions estimate from airborne measurements over a western United States natural gas field. *Geophys. Res. Lett.* 40(16):4393-4397.
8. Peischl J*, et al.* (2018) Quantifying Methane and Ethane Emissions to the Atmosphere From Central and Western U.S. Oil and Natural Gas Production Regions. *J. Geophys. Res.*
9. Ren X*, et al.* (2019) Methane Emissions from the Marcellus Shale in Southwestern Pennsylvania and Northern West Virginia Based on Airborne Measurements. *J. Geophys. Res.* 124(3):1862-1878.
10. Alvarez RA*, et al.* (2018) Assessment of methane emissions from the U.S. oil and gas supply chain. *Science* 361(6398):186-188.
11. Shen L*, et al.* (2022) Satellite quantification of oil and natural gas methane emissions in the US and Canada including contributions from individual basins. *Atmos. Chem. Phys.* 22(17):11203-11215.
